# Peer Victimization, Internalizing Problems, and the Buffering Role of Friendship Quality: Disaggregating Between- and Within-Person Associations

**DOI:** 10.1007/s10964-022-01619-z

**Published:** 2022-04-28

**Authors:** Esther L. Bernasco, Jolien van der Graaff, Wim H. J. Meeus, Susan Branje

**Affiliations:** Department of Youth and Family, Utrecht University, Utrecht, The Netherlands

**Keywords:** Peer victimization, Internalizing problems, Friendship, Adolescence, Peer relations

## Abstract

Although many studies have shown an association between peer victimization and internalizing problems, which may be buffered by friendship quality, it is unclear whether these associations apply to within-person processes as well. This would mean that at times when adolescents experience more victimization than they usually do, they also experience more internalizing problems. The current study disaggregated between- and within-person variation to examine the association between peer victimization and symptoms of depression and anxiety, and the protective effect of friend support and conflict. Participants were 497 Dutch adolescents (56% boys) with a mean age of 13.03 (*SD*_*age*_ = 0.45, ranging from 11.68 to 15.56 at Wave 1). They participated in a 6-wave questionnaire study, with each wave taking place approximately one year after the previous. The results showed that peer victimization was associated with depressive symptoms and anxiety across adolescence, both between and within persons. Friend support buffered this association at the between-person level, but not the within-person level. This study highlights the impact of peer victimization and suggests that friend support may partly protect adolescents from the effects of peer victimization.

## Introduction

Peer victimization and internalizing problems are reciprocally related to each other in a negative cycle. That is, youth who are victimized by peers are more likely to develop depressive and anxious symptoms, and depressed and anxious youth are more likely to become victims of peer victimization (Reijntjes et al., 2010). However, it is unclear whether these effects remain when tested at the intraindividual level, even though theory assumes within-person processes. In other words: Does an individual report more internalizing problems when their experienced level of victimization increases? Furthermore, the negative cycle may be broken by high-quality friendships (Hodges et al., 1999). There is evidence that some aspects of friendship, such as support and protection, may buffer the link between peer victimization and internalizing problems, but the results are mixed. The current study aims to examine associations among between-person differences and within-person change over time in peer victimization and internalizing problems in adolescence, as well as the buffering effect of friendship quality.

### Associations of Peer Victimization with Depressive Symptoms and Anxiety

Peer victimization can be defined as being the target of peers’ behaviors that are intended to be hurtful, either directly or indirectly (Adams et al., 2011). Relational victimization involves acts that hurt the victim’s social relationships, whereas physical victimization involves physical or verbal aggression. Although the two types of victimization show some differences in correlates with other constructs, there is considerable overlap between the two, as many adolescents who experience one type of victimization also experience the other (Casper & Card, 2017). The current study will examine victimization as one construct, including both relational and physical victimization.

Approximately 10–15% of youth are peer victimized (Juvonen & Graham, 2001) and chronic peer victimization has long-lasting effects that may persist into adulthood, including diminished self-esteem, physical and mental health problems, and low-quality relationships (McDougall & Vaillancourt, 2015). Adolescents may be particularly vulnerable to peer victimization compared to children or adults, because peers become increasingly important in adolescence (Bukowski et al., 2011).

Adolescents who reported more peer victimization than others also experienced more internalizing problems, both when assessed concurrently (Hawker & Boulton, 2000) and predictively (Reijntjes et al., 2010). Not only did victimization predict higher levels of internalizing problems, but having high levels of internalizing problems may also put youth at risk for victimization, as these adolescents may be less socially competent and less liked by peers (Christina et al., 2021). However, as these studies showed the link between victimization and internalizing problems at the between-person level, it is not yet clear whether these findings apply to between-person differences only, or also apply to individual change. Controlling for earlier internalizing problems removes some but not all of the between-person variation from the effects. It is unclear whether changes in victimization co-occur with changes in internalizing problems for that same adolescent. This would mean that adolescents are not always stuck in a pattern of victimization and internalizing problems, but change can happen for the better, and that intervening in bullying would help the victim’s mental health.

Although depressive symptoms and anxiety often co-occur within individuals, their associations with peer victimization may differ. Peer victimization is more strongly associated with depressive symptoms than with anxiety (Hawker & Boulton, 2000), and there is some evidence that the link between peer victimization and anxiety is bidirectional, whereas the link between peer victimization and depressive symptoms is not (Sentse et al., 2017). Additionally, positive and negative friendship quality may differentially buffer depressive symptoms and anxiety (Fitzpatrick & Bussey, 2014). It is important to distinguish between depressive symptoms and anxiety when studying the associations of internalizing symptoms with peer victimization and the buffering effect of friendship quality.

The aforementioned studies showing that there are associations between peer victimization and symptoms of depression and anxiety did not explicitly separate between-person associations from within-person associations. Between-person associations concern differences between individuals, whereas within-person associations concern processes of change over time within an individual. To illustrate the difference, consider the association between exercise and heart attacks: People who exercise more frequently are less prone to heart attacks (between-person effect), but an individual is more likely to experience a heart attack while exercising (within-person effect; Curran & Bauer, 2011). In the context of victimization, adolescents who typically experience more victimization than others might also report more internalizing symptoms than others, yet change in victimization for an individual adolescent does not have to be related to change in internalizing symptoms for that same adolescent. When studying only between-person associations, or aggregated between- and within-person associations, it is not possible to draw conclusions on individual change (Hamaker, 2012). Nevertheless, many interventions, which by definition target within-person change, are based on studies that do not separate between- and within-person associations. Although peer victimization may be quite stable for some adolescents, many adolescents experience increases or decreases in peer victimization (Sheppard et al., 2019). Decreases in an individual’s experienced peer victimization may be related to improvements in psychosocial adjustment. For interventions, it is particularly relevant to examine whether an adolescent’s internalizing problems change when peer victimization changes at the individual level. To the authors’ knowledge, this disaggregation of effects has not been done with regard to peer victimization and internalizing problems, yet conclusions are often drawn as if these effects reflect intraindividual processes.

### The Role of Friend Support and Conflict

Close friends are particularly important for wellbeing in adolescence (van der Horst & Coffe, 2012), and peer support interventions have been found to reduce depressive symptoms (Pfeiffer et al., 2011). Friends may form a buffer against the effects of peer victimization rather than affecting the occurrence of victimization per se. The idea that high-quality friendships can protect against the effects of adversity stems from the buffering hypothesis of social support (Cohen & Wills, 1985). Peer victimization threatens the need to belong, but adolescents who experience a sense of belonging within an intimate friendship dyad, may be less affected by the threat that peer victimization may pose. Alternatively, close friends may provide adolescents with social support when faced with adversity (Kendrick et al., 2012). Friendships characterized by high negativity can be a source of stress relating to internalizing problems. Instead of buffering against peer victimization, friendships that are characterized by high levels of conflict may exacerbate its effects on internalizing problems.

Although many different facets of friendship can be distinguished, the current study focuses on friend support and conflict. Support is characterized by mutual trust and reliance on each other, whereas conflict includes negative interactions, such as fights and annoyances within the friendship. A recent systematic review (Schacter et al., 2021) examined the buffering effect of several indices of friendship quality, including constructs such support, friendship self-efficacy and time spent with friends, and the results were ambiguous, even when focusing solely on friend support. Some studies found a buffering effect of friend support (e.g., Cuadros & Berger, 2016; Lim et al., 2011), but others found that victimization was associated with internalizing problems regardless of support (e.g., Brendgen & Poulin, 2018; Davidson & Demaray, 2007), or that the buffering effect was only present for boys (e.g., Cheng et al., 2008; Tanigawa et al., 2011). Some even showed that friend support amplified the association (e.g., Holt & Espelage, 2007; Reid et al., 2016), possibly because close friends tend to use more excessive problem-talk to deal with issues, a phenomenon known as co-rumination (Rose, 2002).

There is less research on friend conflict as a (negative) aspect of friendship quality and its moderating role in the relationship between victimization on internalizing problems. Only one study showed a moderating effect of negative friendship quality: Overall negative (but not positive) friendship quality was associated with a weaker link between victimization and depressive symptoms, but not social anxiety (Fitzpatrick & Bussey, 2014). However, other studies found that conflict did not moderate the effect of victimization on internalizing problems (Hodges et al., 1999), loneliness (Woods et al., 2009), depressive symptoms or social concerns (Schmidt & Bagwell, 2007). In sum, these studies show support for a buffering effect of friend support, and to a lesser extent friend conflict.

Evidence for the buffering effect of friend support has been found at the between-person level: For adolescents who overall report more friend support, for example, smaller between-person associations between victimization and internalizing problems were found. Possibly, this effect also takes place at the within-person level: When an adolescent experiences more friend support or less conflict than usual, they are more resilient against fluctuations in peer victimization. The latter suggests that improving friendship quality may increase adolescents’ resilience.

### Gender differences

Gender may play a role in the associations between peer victimization, friendship quality and internalizing problems, as girls typically score higher on depressive symptoms and anxiety (Graber and Sontag, 2009). Furthermore, there may be gender differences in the association between peer victimization and internalizing problems, because boys and girls handle stressors differently. For example, boys tend to respond more directly to social threats (Underwood & Buhrmester, 2007) and are more likely to externalize in response to social adversity, whereas girls are more likely to internalize (Graber & Sontag, 2009). Girls may also be more likely to (co-)ruminate about the experienced victimization, which in turn may lead to more feelings of depression and anxiety (Starr, 2015), and they tend to be more sensitive to social acceptance, rejection, and support (Rose & Rudolph, 2006).

There is some evidence for gender differences in the association between peer victimization and internalizing problems, as well as in the moderating role of friendship quality. One study showed that boys who were physically victimized reported more loneliness than boys who were not, whereas there was no difference between victimized and non-victimized girls (Woods et al., 2009). Boys and girls may benefit differently from different friendship qualities, depending on the type of victimization they experience. In one study, friends’ help weakened the association of peer victimization with social concerns in girls, whereas it strengthened the association in boys. Similarly, security weakened the association between physical victimization and depressive symptoms in girls, whereas it strengthened the association in boys. However, closeness strengthened the association between physical victimization and depressive symptoms in girls, but not in boys (Schmidt & Bagwell, 2007). Together, these studies showed that the buffering effect of friendship quality may not be the same for boys and girls, for depressive symptoms and anxiety, and for support and conflict.

## Current Study

Previous research showed that adolescents who experienced more peer victimization also experienced more internalizing problems, and that this association may be buffered by friendships, but it is unclear whether these processes also apply to within-person changes in peer victimization, internalizing problems, and friend support and conflict. The current study aimed to add to the literature by examining the buffering effects of friendship quality on the between- and within-person associations between peer victimization and internalizing problems across a six-year period, and by separating different types of internalizing problems (depressive symptoms, anxiety) and friendship quality (support, conflict). The following hypotheses were tested using questionnaire data from six waves of annual assessments among Dutch adolescents in secondary education. First, it was hypothesized that adolescents who experience more peer victimization also experience more internalizing problems. Although this effect has been mostly studied at the between-person level, theory suggests that this association also holds within persons. Therefore, it was expected that when adolescents experience more victimization than usual at one moment they also experience more internalizing problems than usual at that moment. Second, it was hypothesized that the association between peer victimization and internalizing problems is weaker for adolescents with higher-quality friendships, characterized by higher support and lower conflict (between-person). Similarly, it was hypothesized that this association would be weaker at moments when adolescents experienced better friendship quality than usual (within-person). Third, it was hypothesized that the effects of victimization on both depressive symptoms and anxiety as well as the moderating role of friendship quality would be stronger for girls than for boys.

## Methods

### Participants and Procedure

Participants were 497 Dutch adolescents who were followed for six years (from age 13 to 18) as part of the longitudinal RADAR study, which had a full-family design. Adolescents were recruited through 230 participating primary schools in urban areas in the middle and west of the Netherlands. Of the 1544 adolescents and their families who were randomly selected to participate, 1047 were excluded because they did not have two parents and one sibling who was older than 10 years old, which was an exclusion criterion for the original broader project (*n* = 364), no phone records were available (*n* = 99), they refused participation (*n* = 470), or they did not sign informed consent (*n* = 114). This resulted in a sample of 497 adolescents and their best friends (about two to three adolescents per primary school), who enrolled in the study from the first year of secondary school onwards.

Adolescents were asked to participate with their best friend, and it was not required that this best friendship was reciprocated for this study, because adolescents reported on their *perceived* friend support and conflict. However, it is likely they were reciprocated friends (if not reciprocated best friends), as they agreed to participate as friends in the study as well. Most participants reported on the same friend in all waves in which they participated with a friend (55.4%), whereas others reported on a different friend at least once or did not participate with a friend in all waves (44.6%) There were no differences between the group who participated with the same friend and the group who did not on gender, age, or any of the study variables, *p* > 0.119. Because analyses in the current paper required variance within persons, adolescents who only participated in one wave (*n* = 11) or who had only one or zero waves of data on at least one of the study variables (*n* = 15) were excluded from analyses. The excluded participants did not differ from the included participants on gender, age, or any of the six study variables on T1, *p* > 0.273.

The final sample consisted of 471 adolescents (56% boys) with a mean age of 13.03 (*SD* = 0.45, ranging from 11.68 to 15.56) at the first wave. Socio-economic status (SES) in this sample was relatively high, with 88.7% of the families having a medium to high SES score. Across all waves and participants, in 95% of cases adolescents reported on a same-gender friendship, and in 5% of cases they reported on a opposite-gender friendship.

All participants, their parents, and friends (and friends’ parents) gave their informed consent prior to participation. Questionnaires were administered during annual home visits. Participants received €20 for completing each wave. This procedure was approved by the Medical Ethical Review Committee of Utrecht University.

### Missing Data

The attrition rate was relatively low, with at least 88% of the sample participating in each wave. Of the 471 adolescents who participated, 52 (11%) dropped out in total (i.e., did not participate in the 6th wave). Participants who dropped out did not differ from the participants who did not drop out in terms of gender, χ^2^(1) = 1.99, *p* = 0.159, age, *t*(60.69) = 1.85, *p* = 0.069, or the six study variables of interest, *F*(6, 464) = 1.51, *p* = 0.172. Of all participants, 148 (31%) had some missing data. Little’s Missing Completely At Random test showed a normed χ^2^ (χ^2^/df) of 1.21, suggesting that is it unlikely the results were biased due to missing data patterns (Bollen, 1989).

### Measures

#### Peer victimization

Peer victimization was assessed using an adaptation of the Self-report of Aggression and Social Behavior Measure (Linder et al., 2002; Morales & Crick, 1998). For this study, only the two victimization scales were used: Relational Victimization (4 items, e.g., “When others are angry with me, they try to exclude me from joint activities”) and Physical Victimization (3 items, e.g., “Others try to make me do things by physically intimidating me”). Adolescents indicated to what extend the items described their relationship with peers in the past weeks on a scale from 1 (not at all) to 7 (completely true). A peer victimization score was calculated as the average of all items of the Relational Victimization and Physical Victimization subscales. The two subscales were significantly correlated (*r* = 0.58, *p* < 0.001). Cronbach’s alpha showed that reliability was good across waves for the whole scale (0.84 < *α* < 0.87), and was sufficient to good for the Relational Victimization (0.78 < *α* < 0.86) and the Physical Victimization (0.72 < *α* < 0.87) subscales.

#### Depressive symptoms

Depressive symptoms were assessed using a shortened version of the Reynolds Adolescent Depression Scale – 2^nd^ Edition (RADS-2; Reynolds, 2004; Varni et al., 1999). The shortened RADS-2 is a 23-item self-report questionnaire assessing symptoms of dysphoric mood (e.g., “I feel lonely”), negative self-evaluation (“I feel like nobody cares about me”), and somatic pain (e.g., “I am tired”). Adolescents rated the items on a scale from 1 (almost never) to 4 (usually). Depressive symptoms were calculated as the mean of all items. Cronbach’s alpha showed that reliability for the whole scale was excellent across waves (0.91 < α < 0.94).

#### Anxiety symptoms

Anxiety symptoms were assessed using the Child Anxiety-Related Emotional Disorders (SCARED; Birmaher et al., 1997). The SCARED is a 38-item self-report questionnaire that assesses symptoms of somatic complaints or anxiety (e.g., “When I am scared, I have trouble breathing”), school phobia (e.g., “I get a headache or stomachache when I am at school”), social anxiety (e.g., “I feel nervous around people I don’t know very well”), generalized anxiety (e.g., “I am someone who worries a lot”), and separation anxiety (e.g., “I am scared to be home alone”). Adolescents rated the items on a scale from 1 (almost never) to 3 (often). Total anxiety was calculated as the mean of all items. Cronbach’s alpha showed that reliability for the whole scale was excellent across waves (0.91 < *α* < 0.94).

#### Friend support and conflict

Friend support and conflict was assessed using a shortened version of the self-report Network of Relationships Inventory (NRI; Furman & Buhrmester, 1985). This version of the NRI consists of 14 items in two subscales: Support (8 items; e.g., “How often do you share secrets and private feelings with this person?”, “How much does this person treat you like you’re admired and respected?”) and Conflict (6 items, e.g., “How often do you and this person argue with each other?”, “How much do you and this person get on each other’s nerves?”) Adolescents rated to what extent the statements describe the relationship with their closest friend on a scale from 1 (little or not) to 5 (as much as possible). Cronbach’s alpha showed that reliability was good across waves for both the Support (0.86 < *α* < 0.89) and the Conflict (0.87 < *α* < 0.91) subscale.

### Analysis Plan

Hypotheses were tested by fitting a series of multilevel mixed models including disaggregated between- and within-person effects using the “lme4” package in RStudio (Bates, Maechler, Bolker, and Walker, 2015; R Core Team, 2021). Depressive and anxiety symptoms were regressed on victimization, support, conflict, and gender concurrently. This means that associations between outcome and predictor variables were assessed within waves, and no lagged effects were examined. Rather, the longitudinal data were used to assess concurrent associations of individual change in victimization, depressive symptoms and anxiety, and friend support and conflict.

For both depressive symptoms and anxiety, three models were tested and compared: Model 1 included the between- and within-person main effects of victimization, support, conflict, and gender, as well as between- and within-person interactions between victimization and support, and between victimization and conflict; Model 2 included all effects of Model 1 as well as between-person and cross-level interactions between gender and victimization, between gender and support; and between gender and conflict; Model 3 included all effects of Model 2 as well as between-person and cross-level three-way interactions between victimization, support, and gender, and between victimization, conflict and gender. The best model out of the three options was chosen based on model fit and model complexity. More specifically, a model was chosen if at least two out of the four model criteria (AIC, BIC, LL, and deviance) showed it was an improvement of the previous model, and the chi-square showed that the model was a significant improvement to the previous model. Significant interactions were further probed using simple slopes analysis and the Johnson-Neyman technique for region of significance (for noncategorical moderators) to find for which values of the moderator the relationship between the predictor and the independent variable was significant.

To disaggregate between- and within-person effects, predictors (victimization, support, and conflict) were manually split into two components (Bryk & Raudenbush, 1987; Curran & Bauer, 2011). First, person means were calculated by averaging a person’s score across all six waves. This removes all within-person variance across time and reflects between-person effects only. Because there is no variation within individuals in this score, the “lme4” package detects this as a time-invariant predictor. Second, person-mean centered scores per wave were calculated by subtracting an individual’s person mean from that person’s score in a particular wave. These scores reflect a person’s variation around their own mean level across all waves and reflect within-person effects only. Because there is individual variation in this score, the “lme4” package detects this as a time-varying predictor. For each effect in the model, both between-person effects (i.e., the person means; level-2 predictor) and within-person effects (i.e., the person-mean centered scores; level-1 predictor) were entered in the model, except for gender, which is a level-2 predictor. Only unstandardized estimates are reported, because standardization in multilevel models is not without discussion, and the aim was not to study individual variables’ explained variance (e.g., Nezlek, 2001).

## Results

Descriptive statistics are displayed in Table 1. On average, adolescents in the current sample reported low levels of depressive symptoms, anxiety, victimization and conflict, and high levels of support. The intraclass correlations showed that 59% of the variance in depressive and anxious symptoms could be explained by differences between persons, meaning that the other 41% was explained by within-person variation.

**Table 1:** Descriptive statistics for raw scores on all study variables collapsed across all 6 waves

Variable	Descriptive statistics			Bivariate correlations			
	*M*	*SD*	*ICC*	1	2	3	4
1. Depressive symptoms	1.54	0.52	0.59				
2. Anxiety	1.29	0.28	0.60	0.73***			
3. Victimization	1.74	0.87	0.50	0.47***	0.42***		
4. Support	3.35	0.73	0.44	−0.10***	−0.03	−0.08***	
5. Conflict	1.33	0.47	0.37	0.17***	0.14***	0.25***	−0.18***

ICC intraclass correlation

***p < 0.001

For each outcome variable (depressive symptoms or anxiety), three nested models were fit. For both outcome variables, Model 2 (including additional two-way interactions of gender with victimization and friendship quality) was a significant improvement over Model 1. Model 3 (including three-way interactions between victimization, friendship quality, and gender) did not significantly improve model fit, and none of the three-way interactions in any of the Model 3 combinations were significant (see model fit results in Table 2). Results of Model 2 are interpreted for both depressive symptoms and anxiety.

**Table 2:** Model fit information and comparison

Model	Df	AIC	BIC	LL	Deviance	χ^2^
Depressive symptoms as outcome variable						
Model 1	14	2051.16	2132.88	−1011.58	2023.16	
Model 2	20	2019.06	2135.80	−989.53	1979.06	44.09***
Model 3	24	2025.31	2165.40	−988.65	1977.31	1.76
Anxiety as outcome variable						
Model 1	14	−1112.80	−1031.06	570.40	−1140.80	
Model 2	20	−1153.69	−1036.93	596.85	−1193.69	52.90***
Model 3	24	−1147.84	−1007.73	597.92	−1195.84	2.15

For each combination of predictor and outcome variables, Model 1 included main effects of victimization, support, conflict, and gender, and interactions of victimization*support, and victimization*conflict; Model 2 included all effects of Model 1 as well as interactions of gender*victimization, gender*support; and gender*conflict; Model 3 included all effects of Model 2 as well as three-way interactions of victimization*support*gender, and victimization*conflict*gender

***p < 0.001

### The Association between Victimization and Depressive Symptoms

#### Between-person associations

**Fig. 1:**
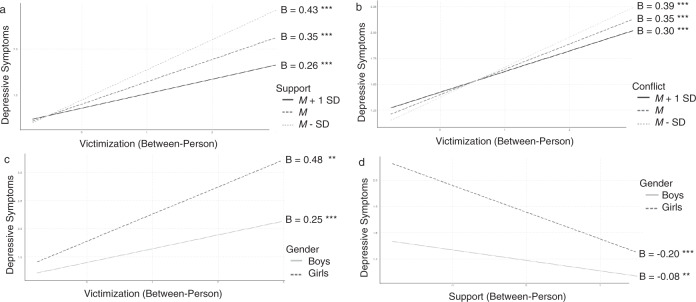
Significant interactions effects with depressive symptoms as outcome variable. **p < 0.01, ***p < 0.001

The significant main effect of gender showed that girls reported more depressive symptoms than boys (see Table 3). The significant main effects of victimization and support on depressive symptoms showed that adolescents who experienced more victimization or less support experienced more depressive symptoms. The positive association between victimization and depressive symptoms was stronger for adolescents with lower levels of friend support than for adolescents with higher levels of friend support, as shown by a significant interaction effect between victimization and support (see Fig. 1a). The Johnson-Neyman procedure showed that the positive association between victimization and depressive symptoms was significant for support levels lower than 4.85 (on a 1-5 scale). The significant interaction between victimization and conflict showed that the positive association between victimization and depressive symptoms was stronger for adolescents with lower levels of conflict, and was significant for conflict levels below 2.66 (on a 1–5 scale; see Fig. 1b). The significant interaction effect between victimization and gender on depressive symptoms showed that the positive association between victimization and depressive symptoms was stronger for girls than for boys, and significant for both (see Fig. 1c). The significant interaction between support and gender showed that the negative association between support and depressive symptoms was stronger for girls than for boys, and significant for both (see Fig. 1d). None of the other between-person associations were significant.

**Table 3:** Model results for the depressive symptoms model

	Between-person associations			Within-person associations		
Variable	Est	SE	CI	Est	SE	CI
Gender	0.38***	0.03	[0.43, 0.32]			
Vict	0.24***	0.03	[0.30, 0.18]	0.14***	0.02	[0.17, 0.11]
Support	−0.09**	0.04	[−0.02, −0.16]	−0.04***	0.01	[−0.02, −0.07]
Conflict	0.08	0.06	[0.18, −0.03]	0.05	0.03	[0.10, −0.01]
Vict*Support	−0.16***	0.04	[−0.08, −0.24]	−0.03	0.03	[0.02, −0.08]
Vict*Conflict	−0.14**	0.06	[−0.02, −0.26]	0.07	0.04	[0.14, −0.01]
Vict*Gender	0.24***	0.05	[0.33, 0.15]	0.03	0.03	[0.08, −0.03]
Support*Gender	−0.12**	0.05	[−0.02, −0.23]			
Conflict*Gender	0.04	0.09	[0.22, −0.14]	0.04	0.05	[0.13, −0.05]

*Vict* victimization. Interactions with gender under “within-person associations” are cross-level interactions. For support*gender, this interaction was not included because there was no significant variation in within-person slopes

*p < 0.05, **p < 0.01, ***p < 0.001

#### Within-person associations

The significant main effects of victimization and support on depressive symptoms showed that at times when adolescents experienced more victimization or less support than usual, they experienced more depressive symptoms than usual. All other within-person associations were nonsignificant.

### The Association between Victimization and Anxiety

#### Between-person associations

**Fig. 2:**
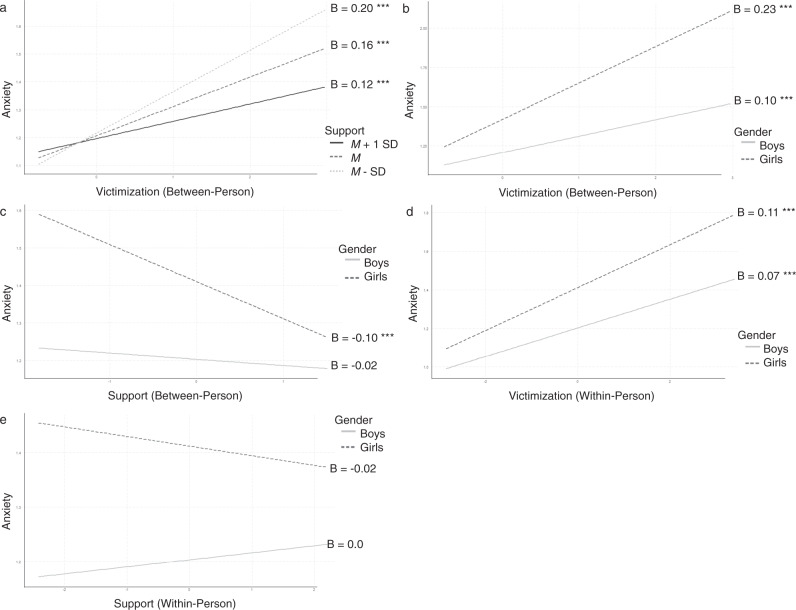
Significant interactions effects with anxiety as outcome variable. ***p < 0.001

The significant main effect of gender showed that girls reported more anxiety than boys (see Table 4). The significant main effects of victimization on anxiety showed that adolescents who experienced more victimization also experienced more anxiety. The positive association between victimization and anxiety was stronger for adolescents with lower levels of support than for adolescents with higher levels of support (see Fig. 2a), as shown by the significant interaction between victimization and support. The Johnson-Neyman procedure showed that the positive association between victimization and anxiety was significant for support levels lower than 4.58 (on a 1–5 scale). The significant interaction between victimization and gender showed that the association between victimization and anxiety was stronger for girls than for boys, and significant for both (see Fig. 2b). Lastly, although there was no significant main effect of support on anxiety, there was a significant interaction between support and gender, revealing a negative association between support and anxiety that was only significant for girls (see Fig. 2c). None of the other between-person associations were significant.

**Table 4:** Model results for the anxiety model

	Between-person associations			Within-person associations		
Variable	Est	SE	CI	Est	SE	CI
Gender	0.21***	0.02	[0.25, 0.18]			
Vict	0.10***	0.02	[0.13, 0.07]	0.07***	0.01	[0.09, 0.05]
Support	−0.02	0.02	[0.02, −0.06]	0.01	0.01	[0.03, −0.01]
Conflict	0.04	0.03	[0.10, −0.02]	0.04**	0.02	[0.07, 0.01]
Vict*Support	−0.08***	0.02	[−0.04, −0.12]	0.00	0.01	[0.03, −0.02]
Vict*Conflict	0.00	0.03	[0.07, −0.07]	0.02	0.02	[0.06, −0.02]
Vict*Gender	0.13***	0.03	[0.18, 0.08]	0.04**	0.02	[0.07, 0.00]
Support*Gender	−0.08**	0.03	[−0.02, −0.14]	−0.03**	0.01	[0.00, −0.06]
Conflict*Gender	0.03	0.05	[0.13, −0.07]	−0.01	0.03	[0.04, −0.06]

Vict victimization. Interactions with gender under “within-person associations” are cross-level interactions

**p < 0.01, ***p < 0.001

#### Within-person associations

The significant main effects of victimization and conflict revealed that at times when adolescents experienced more victimization or conflict than usual, they experienced more anxiety than usual as well. The significant interaction between victimization and gender further revealed that the positive association between victimization and anxiety was stronger for girls than for boys, and significant for both (see Fig. 2d). Lastly, although the main effect of support was not significant, the interaction with gender was. Simples slopes analysis showed that the association between support and anxiety was in opposite directions for boys and girls, and was significant for neither (see Fig. 2e). All other within-person associations were nonsignificant.

### Sensitivity Analysis: Relational and Physical Victimization

Although there was considerable overlap between relational and physical victimization (*r* = 0.58), some studies show that the two types of victimization have different associations with internalizing problems and friendship quality. To explore this possibility, the models were repeated twice: Once for relational and once for physical victimization. Overall, the results were similar to the results of combined victimization (see Online Resource). For depressive symptoms, the model including relational victimization showed no significant main effects for gender or (between- or within-person) support, and no interaction was found between between-person victimization and conflict, or between between-person support and gender. In the model including physical victimization, there was a main effect of between-person conflict, but not support, and there was no interaction of between-person victimization and conflict.

For anxiety, the model including physical victimization showed the same results as the combined model. For relational victimization there was again no significant effect of gender. There was also no interaction of between-person support with gender, but there was a main effect of between-person support. Lastly, there was no interaction effect of within-person relational victimization with gender. Importantly, the between- and within-person main effects of peer victimization and the between-person buffering effect of friend support were stable across different types of victimization.

### Sensitivity Analysis: Controlling for Age

Additionally, analyses were repeated with wave added as a control variable, to check whether the result still hold when accounting for development across adolescence. This did not impact the results (see Online Resource), providing evidence that the associations that were found between constructs at the within-person level are not simply a result of normal development.

It was further explored whether the effects of victimization, support, or conflict changed with age, by including interactions of wave with these variables. This exploration revealed that the within-person effect of victimization on depressive symptoms and anxiety was stronger when adolescents were younger. This means that as adolescents grow older, fluctuations in the victimization they experience do not seem to affect them as much.

### Cross-level Buffering Effects of Friendship Support and Conflict (Exploratory Analysis)

Because the buffering effect of friend support and conflict on the association between peer victimization and internalizing problems was not found within persons, in contrast to the hypotheses, additional analyses were run to explore the post-hoc hypothesis that the effect of within-person variations in peer victimization on internalizing problems could be buffered by long-term friendship quality (i.e., between-person support and conflict). Results showed that none of the cross-level interactions between peer victimization (within-person) and friend support and conflict (between-person) were significant (see Online Resource). This suggests that the within-person associations between adolescents’ peer victimization and internalizing problems do not vary by their overall level of perceived friend support and conflict.

### The Buffering Effect of Friendship Stability (Exploratory Analysis)

In some cases, victimization might happen within a close friend dyad, and it is possible that the effect of support merely reflects that the victimization was not by a close friend. In the current dataset it was not possible to check who the perpetrators of victimization were, but there was information on the friend with whom they participated and whether this friend was the same across waves (friend stability). Friendships characterized by victimization tend to dissolve, so it is likely that victimization occurs in friendships that are not stable across waves rather than in stable friendships. The main models were rerun while controlling for friend stability and included friend stability interactions with victimization, support, and conflict. The interaction effect of friend stability with victimization was significant, suggesting that the effect of victimization on both depressive and anxiety symptoms was stronger for adolescents who did not have a stable friendship (see Online Resource). Importantly, the buffering effect of friend support remained significant after controlling for main and interaction effects of friend stability, indicating that stability does not fully explain the effect of friend support.

## Discussion

Previous research has shown an association of peer victimization with depressive symptoms and anxiety, which may be buffered by friendship. However, it is unclear whether these findings also apply to within-person processes. The current study disaggregated between- and within-person variation to show that the association between peer victimization and both depressive symptoms and anxiety exists on both between- and within-person level, whereas the buffering effect of friend support only applies to between-person variation.

### Between- and Within-Person Effects of Peer Victimization on Internalizing Problems

The current study expanded on previous studies by examining to what extent variability could be attributed to differences in average levels of predictors (between-person effects) and to individuals’ year-to-year fluctuations (within-person effects). Between-person results showed that adolescents who experienced more victimization on average also experienced more depressive symptoms and anxiety, replicating meta-analyses showing both concurrent (Hawker & Boulton, 2000) and predictive (Christina et al., 2021) associations. Peer victimization may affect internalizing problems by increasing feelings of loneliness and self-doubt, lowering self-esteem, or frustrating the need to belong (Baumeister & Leary, 1995). Within-person results showed a similar pattern: At moments when adolescents experienced more victimization, they also experienced more depressive symptoms and anxiety. This means that adolescents are not stuck in a pattern of victimization and internalizing problems, but individual change is possible. Indeed, when an adolescent’s peer victimization levels decrease, internalizing symptoms may also decrease. Sensitivity analyses showed that these results were consistent when controlling for age and when analyzing relational and physical victimization separately, highlighting the robustness of the association. It is important to note that although internalizing problems are analytically presented as the outcome variable, the current study cannot draw conclusions about the directions of the effect. The main aim was not to study the predictive bidirectional effects, but rather to study individual change and to separate the between- and within-person associations in one model.

### Friend Support as a Buffer Against the Association Between Peer Victimization and Internalizing Problems

The current study revealed evidence for the hypothesized between-person buffering effect of friendship support on the link between peer victimization and symptoms of depression and anxiety, in line with previous studies (Schacter et al., 2021). Adolescents who experience more support from their friends seem to suffer less from the effects of peer victimization. Positive friendships may buffer the effect of peer victimization because they may provide a stronger sense of belonging (Baumeister & Leary, 1995), or provide the emotional support needed to deal with stressful situations (Kendrick et al., 2012).

Friend conflict, in contrast, did not moderate the association between peer victimization and symptoms of depression and anxiety, suggesting that the strength of the association between victimization and internalizing problems is not exacerbated by the level of conflict within a friendship. Possibly, the presence of support within a friendship is more important in the context of peer victimization than the absence of conflict. Alternatively, the negative effect of peer victimization is not as important as the presence of high levels of negativity within close friendship. Furthermore, some level of conflict may be normative, and relatively high levels of conflict may not necessarily be an exacerbating factor. These findings also highlight the idea that friendship quality consists of distinct underlying constructs and researchers should study its components separately.

The buffering effect of friend support did not hold within persons. This suggests that the buffering effect of friend support is not sensitive to temporary changes in support. Moreover, sensitivity analyses using cross-level interactions showed that adolescents’ general level of friend support did not make adolescents less sensitive to variations in peer victimization. Having a supportive friend may thus protect against the effects of adolescents’ general victimization status, but not against individuals’ increases in experienced victimization.

### The Role of Gender in the Link Between Peer Victimization and Internalizing Problems

In line with previous research (Graber & Sontag, 2009), it was found that girls reported more depressive symptoms and anxiety than boys. Gender differences were also tested with regard to the association of peer victimization with depressive symptoms and anxiety, because boys and girls respond differently to (social) adversity, stress, and support (e.g., Rose & Rudolph, 2006). As hypothesized, the (between-person) associations of peer victimization with internalizing problems were stronger for girls than for boys.

In contrast to the hypothesis, none of the three-way interactions between victimization, friendship quality, and gender was significant or improved model fit. This suggests that the buffering effect of friendship quality is similar for boys and girls. However, it is worth noting that there was relatively little power for three-way interactions, so this result should be considered with care.

### Strengths, Limitations, and Future Research

An important limitation of current study is that it relied only on self-report measures and these may be biased. For example, depression is associated with negative cognitive biases (Platt et al., 2017) which may cause adolescents who scored higher on depressive symptoms to also perceive their friendships or peer victimization more negatively than their friends or a peer-reported measure would. This reporter-bias may have inflated the associations between peer victimization and internalizing problems. However, it can be argued that internalizing problems are an internal process that cannot be easily assessed by others, in contrast to externalizing problems, for example, and there is evidence that the link between social support and depression is based on more than just biases (Cutrona, 1989). Another limitation is the relatively homogenous sample. Participants were Dutch and the majority had mid-to-high SES. There were relatively few participants from a low-SES background in the current sample. This may be related to the inclusion criteria that were applied for purposes of the broader project, such as two-parent households with at least one sibling. Although it is likely that findings apply to some other populations, such as single-parent or only-child households, it is possible that some findings differ. Adolescents from ethnic minority backgrounds or low SES may experience different kinds of victimization, including discrimination, and may be more at risk for victimization too. The links with internalizing problems or friendship quality may be different for them. Relatedly, the majority of studies on this topic stem from North America and Europe, and it is unclear how findings extend to non-Western cultures. Future studies should focus on recruiting participants from underrepresented populations, such as ethnic minorities and non-Western countries, to examine cross-cultural differences and similarities. Lastly, the dataset did not include information on the perpetrators of the victimization. Victimization can occur within friendship groups or dyads, and it is possible that some of the buffering effect of friend support is due to a lack of dyadic victimization, although the sensitivity analysis showed it likely does not play a large role. Even within friendships that include dyadic victimization, both positive and negative friendship quality can be quite high (Daniels et al., 2010). Studies that include more information on perpetrators of victimization can shed more light on the associations between friendship quality, dyadic victimization, and other types of victimization.

This study also had some strengths that increased the contribution of this paper to the literature in several ways. First, to the authors’ knowledge, it is the first study to disaggregate the between- and within-person effects of peer victimization on depressive and anxious symptoms in adolescents. In doing so, the current study was able to show that intra-individual fluctuations in peer victimization are associated with fluctuations in internalizing problems, thereby showing the relevance of intervening on either of these issues for individual wellbeing. Second, this study assessed different aspects of friendship quality, of internalizing problems, and of peer victimization. This revealed stable effects across depressive symptoms and anxiety, and across relational and physical victimization, but that it is important to separately examine support and conflict within a friendship, because they did not show the same moderating effect.

It is yet unclear how improving friend support in individuals may buffer the effects of peer victimization on the long-term. Since this study’s aims were related to associations within one time point, rather than prediction, it was not possible to draw conclusions about short- and long-term effects. Future research should further disentangle the mechanisms behind the buffering effect of friend support.

## Conclusion

The association between peer victimization and internalizing problems may be buffered by friendship quality, but it was unclear whether these effect also take place within individuals. This study disaggregated between-person effects (individual average) from within-person effects (variation around individual average) over 6 waves of annual assessments. Results showed both between- and within-person effects of victimization on internalizing problems, but only between-person buffering effects of friend support. This means that when an adolescent experiences increases in victimization, they also experience increases in depressive symptoms and anxiety, regardless of their level of friend support. These findings suggest that intervening in victimization might be a useful way to decrease symptoms of depression and anxiety in individuals. This paper also highlights that between-person findings may mirror within-person effects, but this is not always the case.

## Supplementary Information

Online Supplementary Materials
