# Supplementary material for: Peer Victimization, Internalizing Problems, and the Buffering Role of Friendship Quality: Disaggregating Between- and Within-Person Associations
Source: J Youth Adolesc. 2022 Apr 28;51(8):1653–66. doi: 10.1007/s10964-022-01619-z (PMC9232402; doi:10.1007/s10964-022-01619-z)

Online Supplementary Materials

Paper title	Peer Victimization, Internalizing Problems, and the Buffering Role of Friendship Quality: Disaggregating Between- and Within-Person Associations
Journal	Journal of Youth and Adolescence
Authors	Esther L. Bernasco (corresponding author)<br>Jolien van der Graaff<br>Wim H. J. Meeus<br>Susan Branje

S1. Sensitivity Analysis: Separating Relational and Physical Victimization

Table S1

Descriptive Statistics for Physical and Relational Victimization Collapsed Across all 6 Waves

				Bivariate correlations				
Variable	*M*	*SD*	ICC	Depression	Anxiety	Support	Conflict	RelVic
3. RelVic	1.95	1.07	0.51	.49 ***	.45 ***	-.05 *	.22 ***	
4. PhyVic	1.45	0.81	0.40	.29 ***	.25 ***	-.10 ***	.23 ***	.58 ***

Note. ICC = intraclass correlation, RelVic = relational victimization, PhyVic = physical victimization.

* $p < .05$. *** $p < .001$.

Table S2

Model Results for Relational Victimization

	Between-person associations			Within-person associations		
Variable	Est	SE	95% CI	Est	SE	95% CI
			Depressive symptoms			
Gender	0.31 ***	0.03	[0.37, 0.25]			
RelVic	0.21 ***	0.03	[0.26, 0.16]	0.12 ***	0.01	[0.14, 0.09]
Support	-0.09 **	0.04	[-0.02, -0.16]	-0.04 ***	0.01	[-0.02, -0.07]
Conflict	0.08	0.06	[0.19, -0.03]	0.05	0.03	[0.11, 0.00]
RelVic*Support	-0.11 ***	0.03	[-0.05, -0.17]	-0.02	0.02	[0.02, -0.06]
RelVic*Conflict	-0.08	0.05	[0.02, -0.19]	0.05	0.03	[0.11, -0.01]
RelVic*Gender	0.12 ***	0.04	[0.19, 0.05]	0.00	0.02	[0.05, -0.04]
Support*Gender	-0.09	0.06	[0.02, -0.20]			
Conflict*Gender	0.07	0.09	[0.25, -0.11]	0.03	0.05	[0.12, -0.06]
			Anxiety			
Gender	0.18 ***	0.02	[0.21, 0.15]			
RelVic	0.09 ***	0.01	[0.12, 0.06]	0.06 ***	0.01	[0.08, 0.04]
Support	-0.02	0.02	[0.02, -0.06]	0.01	0.01	[0.03, 0.00]
Conflict	0.05	0.03	[0.11, -0.01]	0.05 ***	0.02	[0.08, 0.02]
RelVic*Support	-0.05 ***	0.02	[-0.02, -0.09]	0.00	0.01	[0.02, -0.02]
RelVic*Conflict	0.01	0.03	[0.07, -0.04]	0.01	0.02	[0.04, -0.02]
RelVic*Gender	0.07 ***	0.02	[0.11, 0.03]	0.02	0.01	[0.05, 0.00]
Support*Gender	-0.07 **	0.03	[-0.01, -0.13]	-0.03 **	0.01	[0.00, -0.06]
Conflict*Gender	0.02	0.05	[0.12, -0.08]	-0.02	0.02	[0.03, -0.07]

Note. RelVic = relational victimization. Boys = 0, girls = 1. Interactions with gender under "within-person associations" are cross-level interactions. When there was no variation in within-person slopes, these interactions were not included.

** $p < .01$, *** $p < .001$.

Table S3

Model Results for Physical Victimization

	Between-person associations			Within-person associations		
Variable	Est	SE	95% CI	Est	SE	95% CI
			Depressive symptoms			
Gender	0.49 ***	0.04	[0.56, 0.43]			
PhyVic	0.22 ***	0.04	[0.29, 0.15]	0.10 ***	0.02	[0.14, 0.07]
Support	-0.08 **	0.04	[0.00, -0.16]	-0.04 ***	0.01	[-0.01, -0.06]
Conflict	0.11	0.06	[0.23, -0.02]	0.05	0.03	[0.11, 0.00]
PhyVic*Support	-0.16 **	0.06	[-0.05, -0.28]	-0.02	0.03	[0.03, -0.07]
PhyVic*Conflict	-0.15	0.08	[0.01, -0.31]	0.06	0.04	[0.13, -0.01]
PhyVic*Gender	0.33 ***	0.07	[0.46, 0.21]	0.02	0.03	[0.08, -0.04]
Support*Gender	-0.18 ***	0.06	[-0.06, -0.31]			
Conflict*Gender	0.07	0.11	[0.27, -0.14]	0.06	0.05	[0.16, -0.03]
			Anxiety			
Gender	0.26 ***	0.02	[0.30, 0.23]			
PhyVic	0.09 ***	0.02	[0.13, 0.06]	0.06 ***	0.01	[0.07, 0.04]
Support	-0.01	0.02	[0.03, -0.06]	0.01	0.01	[0.03, -0.01]
Conflict	0.04	0.04	[0.11, -0.03]	0.04 **	0.02	[0.08, 0.01]
PhyVic*Support	-0.09 **	0.03	[-0.03, -0.15]	0.00	0.01	[0.03, -0.03]
PhyVic*Conflict	0.00	0.04	[0.09, -0.09]	0.02	0.02	[0.06, -0.02]
PhyVic*Gender	0.15 ***	0.04	[0.22, 0.08]	0.02	0.02	[0.05, -0.01]
Support*Gender	-0.11 ***	0.04	[-0.04, -0.18]	-0.02	0.01	[0.01, -0.05]
Conflict*Gender	0.10	0.06	[0.21, -0.02]	0.01	0.03	[0.06, -0.04]

Note. PhyVic = physical victimization. Boys = 0, girls = 1. Interactions with gender under "within-person associations" are cross-level interactions. When there was no variation in within-person slopes, these interactions were not included.

** $p < .01$, *** $p < .001$.

S2. Sensitivity Analysis: Controlled for Time

Table S4

Model Results Controlled for Age

	Between-person associations			Within-person associations		
Variable	Est	SE	95% CI	Est	SE	95% CI
Depressive symptoms						
Time	0.01 **	0.00	[0.01, 0.00]			
Gender	0.38 ***	0.03	[0.43, 0.32]			
Victimization	0.24 ***	0.03	[0.30, 0.18]	0.14 ***	0.02	[0.18, 0.11]
Support	-0.09 **	0.04	[-0.02, -0.16]	-0.04 ***	0.01	[-0.01, -0.06]
Conflict	0.08	0.06	[0.18, -0.03]	0.05	0.03	[0.10, -0.01]
Vict*Support	-0.16 ***	0.04	[-0.08, -0.24]	-0.03	0.03	[0.02, -0.08]
Vict*Conflict	-0.14 **	0.06	[-0.02, -0.26]	0.07	0.04	[0.14, -0.01]
Vict*Gender	0.24 ***	0.05	[0.33, 0.15]	0.03	0.03	[0.08, -0.03]
Support*Gender	-0.12 **	0.05	[-0.02, -0.23]			
Conflict*Gender	0.04	0.09	[0.22, -0.14]	0.04	0.05	[0.13, -0.05]
Anxiety						
Time	-0.01 ***	0.00	[0.00, -0.01]			
Gender	0.21 ***	0.02	[0.25, 0.18]			
Victimization	0.10 ***	0.02	[0.13, 0.07]	0.07 ***	0.01	[0.09, 0.05]
Support	-0.02	0.02	[0.02, -0.06]	0.01	0.01	[0.03, -0.01]
Conflict	0.04	0.03	[0.10, -0.02]	0.04 **	0.02	[0.07, 0.01]
Vict*Support	-0.08 ***	0.02	[-0.04, -0.12]	0.00	0.01	[0.03, -0.02]
Vict*Conflict	0.00	0.03	[0.07, -0.07]	0.02	0.02	[0.06, -0.02]
Vict*Gender	0.13 ***	0.03	[0.18, 0.08]	0.03 **	0.02	[0.07, 0.00]
Support*Gender	-0.09 **	0.03	[-0.03, -0.15]	-0.03 **	0.01	[0.00, -0.06]
Conflict*Gender	0.03	0.05	[0.13, -0.07]	-0.01	0.02	[0.04, -0.06]

Note. Vict = victimization. Interactions with gender under "within-person associations" are cross-level interactions. When there was no variation in within-person slopes, these interactions were not included.

** $p < .01$, *** $p < .001$.

Table S5

Model Results Controlled for Age Including Interactions

Variable	Between-person			Within-person		
	Est	SE	95% CI	Est	SE	95% CI
			Depressive symptoms			
Gender	0.37 ***	0.03	[0.43, 0.31]			
Vict	0.24 ***	0.03	[0.30, 0.19]	0.27 ***	0.03	[0.33, 0.21]
Support	-0.08 **	0.04	[-0.01, -0.15]	-0.05	0.03	[0.01, -0.11]
Conflict	0.08	0.06	[0.19, -0.03]	0.02	0.05	[0.13, -0.08]
Vict*Support	-0.15 ***	0.04	[-0.08, -0.23]	-0.06 **	0.03	[0.00, -0.11]
Vict*Conflict	-0.13 **	0.06	[-0.01, -0.26]	0.05	0.04	[0.13, -0.02]
Gender*Vict	0.24 ***	0.05	[0.32, 0.15]	0.03	0.03	[0.08, -0.02]
Gender*Support	-0.12 **	0.05	[-0.02, -0.23]			
Gender*Conflict	0.04	0.09	[0.22, -0.14]	0.03	0.04	[0.12, -0.06]
Age				0.01	0.00	[0.01, 0.00]
Age*Vict				-0.04 ***	0.01	[-0.02, -0.06]
Age*Support				0.00	0.01	[0.02, -0.01]
Age*Conflict				0.01	0.01	[0.03, -0.02]
			Anxiety			
Gender	0.21 ***	0.02	[0.24, 0.17]			
Vict	0.10 ***	0.02	[0.13, 0.07]	0.13 ***	0.02	[0.17, 0.10]
Support	-0.02	0.02	[0.02, -0.06]	0.03	0.02	[0.06, 0.00]
Conflict	0.04	0.03	[0.10, -0.02]	0.03	0.03	[0.09, -0.02]
Vict*Support	-0.08 ***	0.02	[-0.03, -0.12]	-0.01	0.01	[0.02, -0.04]
Vict*Conflict	0.00	0.03	[0.07, -0.06]	0.01	0.02	[0.05, -0.03]
Gender*Vict	0.13 ***	0.03	[0.18, 0.08]	0.03 **	0.02	[0.07, 0.00]
Gender*Support	-0.09 **	0.03	[-0.02, -0.15]	-0.03 **	0.01	[0.00, -0.06]
Gender*Conflict	0.03	0.05	[0.13, -0.07]	-0.01	0.02	[0.03, -0.06]
Age				-0.01 ***	0.00	[-0.01, -0.01]
Age*Vict				-0.02 ***	0.00	[-0.01, -0.03]
Age*Support				-0.01	0.00	[0.00, -0.01]
Age*Conflict				0.00	0.01	[0.02, -0.01]

Note. Vict = victimization. Interactions with gender under “within-person associations” are cross-level interactions. For support*gender, this interaction was not included because there was no significant variation in within-person slopes.

* $p < .05$, ** $p < .01$, *** $p < .001$.

Figure 1

The Moderating Role of Age on the Effect of Victimization on Depressive Symptoms (Left) and Anxiety (Right)

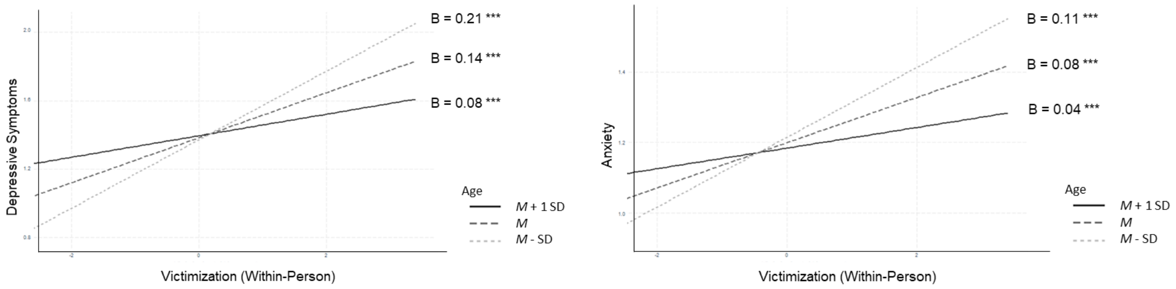

S3. Sensitivity Analysis: Cross-Level Interactions

Table S6

Model Results with Cross-Level Interactions Between Friendship Quality (Within-Person) and Peer Victimization (Between-Person)

	Between-person associations			Within-person associations		
Variable	Est	SE	95% CI	Est	SE	95% CI
			Depressive symptoms			
Gender	0.37 ***	0.03	[0.42, 0.31]			
Victimization	0.26 ***	0.03	[0.31, 0.20]	0.14 ***	0.02	[0.18, 0.10]
Support	-0.08 **	0.04	[-0.01, -0.15]			
Conflict	0.07	0.06	[0.18, -0.04]			
Vict*Support	-0.14 ***	0.04	[-0.06, -0.22]	-0.02	0.03	[0.03, -0.07]
Vict*Conflict	-0.16 **	0.06	[-0.04, -0.28]	-0.06	0.04	[0.02, -0.13]
Vict*Gender	0.22 ***	0.04	[0.30, 0.13]	0.05	0.03	[0.11, -0.01]
Support*Gender	-0.13 **	0.05	[-0.02, -0.24]			
Conflict*Gender	0.02	0.09	[0.20, -0.15]			
			Anxiety			
Gender	0.21 ***	0.02	[0.24, 0.17]			
Victimization	0.12 ***	0.02	[0.15, 0.08]	0.08 ***	0.01	[0.10, 0.06]
Support	-0.01	0.02	[0.03, -0.05]			
Conflict	0.03	0.03	[0.09, -0.03]			
Vict*Support	-0.05 **	0.02	[-0.01, -0.09]	0.00	0.01	[0.03, -0.03]
Vict*Conflict	-0.03	0.03	[0.04, -0.10]	-0.01	0.02	[0.03, -0.06]
Vict*Gender	0.11 ***	0.03	[0.16, 0.06]	0.04 **	0.02	[0.07, 0.01]
Support*Gender	-0.10 ***	0.03	[-0.03, -0.16]			
Conflict*Gender	0.01	0.05	[0.11, -0.09]			

Note. Vict = victimization. All interactions under “within-person associations” are cross-level interactions.

** p < .01, *** p < .001.

S4. Ad-Hoc Analysis: Friend Stability

Table S7

Model results controlling for friend stability

Variable	Between-person			Within-person		
	Est	SE	95% CI	Est	SE	95% CI
			Depressive symptoms			
Stability	0.00	0.03	[0.06, -0.05]			
Gender	0.37 ***	0.03	[0.43, 0.31]			
Vict	0.18 ***	0.04	[0.25, 0.11]	0.12 ***	0.02	[0.16, 0.08]
Support	-0.08	0.05	[0.01, -0.17]	-0.03 **	0.02	[0.00, -0.06]
Conflict	0.07	0.07	[0.21, -0.08]	0.03	0.03	[0.09, -0.04]
Vict*Support	-0.15 ***	0.04	[-0.08, -0.23]	-0.03	0.03	[0.02, -0.08]
Vict*Conflict	-0.10	0.06	[0.03, -0.22]	0.07	0.04	[0.14, 0.00]
Gender*Vict	0.24 ***	0.05	[0.33, 0.15]	0.03	0.03	[0.08, -0.02]
Gender*Support	-0.13 **	0.06	[-0.02, -0.23]			
Gender*Conflict	0.02	0.09	[0.20, -0.15]	0.04	0.05	[0.13, -0.05]
Stability*Vict	0.14 ***	0.04	[0.23, 0.05]	0.04	0.03	[0.09, -0.01]
Stability*Support	0.01	0.05	[0.11, -0.09]	-0.03	0.03	[0.02, -0.08]
Stability*Conflict	0.03	0.09	[0.21, -0.14]	0.04	0.05	[0.13, -0.05]
			Anxiety			
Stability	-0.02	0.02	[0.02, -0.05]			
Gender	0.21 ***	0.02	[0.24, 0.17]			
Vict	0.08 ***	0.02	[0.12, 0.04]	0.07 ***	0.01	[0.10, 0.05]
Support	-0.01	0.03	[0.04, -0.06]	0.01	0.01	[0.03, -0.02]
Conflict	0.05	0.04	[0.13, -0.03]	0.04 **	0.02	[0.08, 0.00]
Vict*Support	-0.08 ***	0.02	[-0.04, -0.12]	0.00	0.01	[0.03, -0.02]
Vict*Conflict	0.01	0.04	[0.08, -0.06]	0.02	0.02	[0.06, -0.02]
Gender*Vict	0.13 ***	0.03	[0.18, 0.08]	0.04 **	0.02	[0.07, 0.00]
Gender*Support	-0.09 **	0.03	[-0.03, -0.15]	-0.03 **	0.01	[0.00, -0.06]
Gender*Conflict	0.02	0.05	[0.12, -0.08]	-0.01	0.02	[0.04, -0.06]
Stability*Vict	0.05 **	0.02	[0.10, 0.00]	0.01	0.02	[0.04, -0.03]
Stability*Support	-0.01	0.03	[0.05, -0.06]	0.02	0.01	[0.04, -0.01]
Stability*Conflict	-0.01	0.05	[0.08, -0.11]	0.01	0.03	[0.06, -0.04]

Note. Vict = victimization. Interactions with gender under "within-person associations" are cross-level interactions. For support*gender, this interaction was not included because there was no significant variation in within-person slopes.

* $p < .05$, ** $p < .01$, *** $p < .001$.

Figure 2

The Moderating Role of Friend Stability on the Effect of Victimization on Depressive Symptoms (Left) and Anxiety (Right)

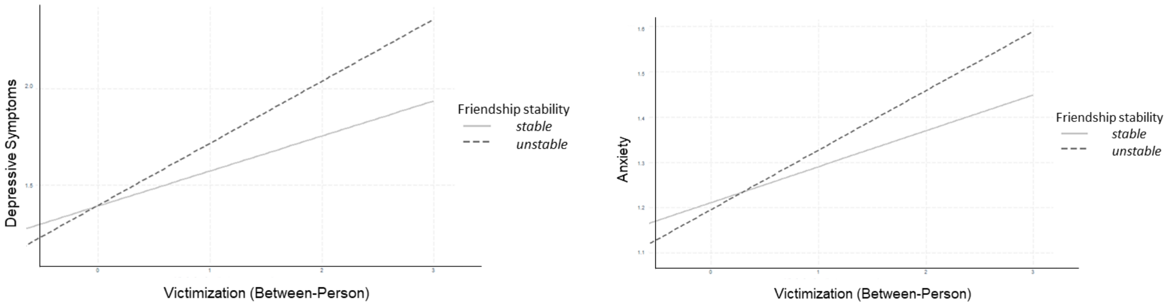

Supplement: Supplementary file 1 — Online Supplementary Materials [file 10964_2022_1619_MOESM1_ESM.pdf]
